# Aberrant p53 immunostaining patterns in breast carcinoma of no special type strongly correlate with presence and type of *TP53* mutations

**DOI:** 10.1007/s00428-024-03897-3

**Published:** 2024-08-27

**Authors:** Hannes Armbruster, Tilman Schotte, Isabell Götting, Mathis Overkamp, Massimo Granai, Lea Louise Volmer, Veronika Bahlinger, Sabine Matovina, André Koch, Dominik Dannehl, Tobias Engler, Andreas D. Hartkopf, Sara Y. Brucker, Irina Bonzheim, Falko Fend, Annette Staebler, Ivonne Montes-Mojarro

**Affiliations:** 1 Institute of Pathology and Neuropathology and Comprehensive Cancer Center Tübingen, Eberhard-Karls-University, Liebermeisterstraße 8, 72076 Tübingen, Germany; 2 Department of Woman’s Health, Eberhard-Karls-University, Tübingen, Germany

**Keywords:** Breast cancer, P53, Immunohistochemistry, *TP53* mutation, *PIK3CA*, NGS

## Abstract

Recent studies have revealed an association between *TP53* mutations and endocrine resistance in hormone receptor-positive, HER2-negative breast cancer (HR + HER2 −BC). Aberrant p53 immunostaining (IHC) patterns may provide a surrogate marker for *TP53* mutations. Building upon a ternary algorithm of aberrant staining patterns, this study evaluates the reliability of p53 IHC as screening tool for *TP53* mutations in BC (NST). Furthermore, it describes the histopathological and molecular characteristics of *TP53*-mutated cases, along with the mutational status of *PIK3CA*. This study comprised 131 early-stage, node-negative BCs with available core biopsies and resection specimens. Cases were categorized as follows: HR + HER2 − (85 cases), HER2 + (21 cases) and triple negative (TN, 25 cases). Aberrant IHC staining patterns for p53 were defined as overexpression (OE), complete absence (CA) and cytoplasmic (CY). In addition, targeted sequencing of *TP53* and *PIK3CA* genes was performed. *TP53* mutations were identified in 53 of 126 cases (42.1%). Within HR + HER2 − cases, *TP53* mutations were found in 17 of 80 cases (21.3%). IHC accurately predicted *TP53* mutation in 96.2% of cases with a specificity of 100%. Additionally, there was a significant agreement between missense mutations and OE, as well as between truncating mutations and CA (κ 73% and 76%). CY was observed in two TN cases with truncating mutations within the nuclear localization signalling domain of p53. *TP53*-mutated cases exhibited higher grade, greater nuclear pleomorphism and higher Ki-67 proliferation index and were associated with the *PIK3CA* wild-type status (*p* < 0.001). p53 IHC may provide a useful screening tool for identifying *TP53*-mutated BC of NST.

**Supplementary Information:**

The online version contains supplementary material available at 10.1007/s00428-024-03897-3.

## Introduction

Mutations in the tumor suppressor gene *TP53* can be identified in approximately 20–40% of all breast carcinomas (BCs) with different frequencies in the established molecular subtypes [2, 8]. *TP53* mutations have been predominantly associated with basal-like tumors, but also occur in HER2 + and in luminal-like (HR + HER2 −) BCs [1, 9]. Mostly because of its dominant association with basal-like carcinoma, this genetic alteration has been associated with decreased survival in metastatic BC [21]. The *TP53*-encoded protein p53 is known to play an important role in cell cycle arrest, regulation of apoptosis, the response to genotoxic stress and DNA alterations [26, 31]. *TP53* mutations result in genetic instability with increased somatic mutations, unbalanced DNA copy number variations and multiple chromosomal alterations [19].

Clinical studies have demonstrated that *TP53* mutations are associated with poor prognosis also in HR + HER2 −BC [11, 21]. This can in part be attributed to the significant correlation between *TP53* mutations in HR + HER2 −BC and resistance to endocrine therapies, including antiestrogens and aromatase inhibitors [13]. Recently, the analysis of a cohort of primary tumors treated with preoperative short-term endocrine therapy showed a reduced response rate to endocrine therapy in cases with *TP53* mutations, as measured by insufficient decrease of the proliferation marker Ki-67 in the resection specimen compared to the diagnostic core biopsy [12]. More recently, *TP53* mutations have also been identified as an independent factor, contributing to the high 21-gene recurrence score [15]. In conclusion, patients carrying *TP53* mutations in early HR + HER2 −BC are more likely to experience recurrence, distant metastasis and shorter overall survival under adjuvant endocrine therapy [33].

Based on these findings, evaluation of the *TP53* status might influence therapeutic decisions in HR + HER2 −BC in the future. However, *TP53* mutation analysis is currently not part of the standard diagnostic workup outside of clinical studies, and risk profiling beyond standard clinical-pathological parameters relies primarily on commercial gene expression profiling. Given the potential future role of *TP53* status and the cost and technical effort of mutational profiling, a fast and broadly available screening tool for *TP53* alterations is desirable [33]. In the last decade, p53 IHC has become an accepted surrogate marker for *TP53* status in other tumor entities such as endometrial or ovarian cancer [17, 28, 32]. Major progress in the molecular classification of these tumors in the clinical setting was enabled by a robust and reproducible algorithm to interpret patterns of p53 staining rather than relying on a cutoff for overexpression of p53 alone [17]. These aberrant patterns include overexpression (OE), defined as continuous band-like pattern of strongly stained tumor cell nuclei (mostly associated with missense mutations), complete absence (CA, resulting from truncating mutations) or cytoplasmic (CY) expression (underlying truncating mutations with disturbance of the nuclear localization signal), whereas the wild-type pattern shows variable levels of nuclear expression in the tumor cell population. This approach has enabled the integration of IHC as a standard diagnostic marker for the “p53 abnormal” molecular subtype in endometrial cancer, which is associated with high-risk disease [5].

However, the clinical significance of immunohistochemical pattern analysis of p53 expression in BC remains uncertain. A previous study demonstrated that p53 staining patterns have an adverse effect on survival when a bimodal distribution with extreme negative and extreme positive staining is considered [3]. In our study, we evaluated whether a more comprehensive evaluation of p53 staining patterns can predict the mutational status of *TP53* in BC, including the consideration of the CY pattern as an additional form of aberrant expression. We aimed to correlate these patterns with specific types of mutations and to explore the characteristics of *TP53*-mutated cases by correlating *TP53* mutational status with histomorphological and molecular features including tumor-grade, therapy-relevant subtype and *PIK3CA* mutational status.

## Materials and methods

### Patients, samples and clinical data

This study retrospectively analyzed formalin-fixed paraffin-embedded (FFPE) tumor tissue samples from the Institute of Pathology and Neuropathology (Tübingen University Hospital). A total of 131 consecutive cases of female patients diagnosed with early-stage BC of non-special type (NST) (pT1-3 unifocal, N0M0) at our institution between 2010 and 2012 with available core biopsies and resection specimens without neoadjuvant treatment were collected. As part of routine workup, cases had been immunophenotyped for hormone receptors, proliferation rate (Ki-67) and HER2 status. Fluorescence in situ hybridization (FISH) had been performed according to the ASCO/CAP guidelines on cases with HER2 Score 2 + [36]. All biopsies and resection specimens were re-evaluated histologically on hematoxylin and eosin (H&E)–stained slides by two experienced breast pathologists (A. S. and I. A. M-M) for Elston and Ellis score. For assessment of biological subtype, immunostains were re-evaluated and proliferation rate was calculated according to the recommendations of the Ki-67 Working Group [7]. Medical records were retrieved, including age, previous medical history and follow-up. This study was performed according to the Declaration of Helsinki and was approved by the Ethics Committee of the Medical Faculty of the University of Tübingen (547/2021BO2).

### p53 immunohistochemistry

IHC for p53 (DO-7, Dilution 1:400, Novocastra, Leica Biosystems, Wetzlar, Germany) was performed using an automated stainer (Ventana Medical Systems, Tucson, Arizona, USA) in accordance with the manufacturer’s protocol. Assessment of p53 staining was carried out both on resection specimens and on core biopsies, independently by two pathologists following the recent recommendations for gynecological neoplasms, with aberrant staining defined as OE, CA and CY [17, 28]. In case of disagreement, stainings were re-evaluated by another senior pathologist (F.F.) to reach consensus.

### DNA isolation

To enrich tumor cell content, tumors of the resection specimens were macroscopically dissected, and tumor cell content was estimated proportionally. Genomic DNA was extracted from macrodissected 5 µm paraffin sections using the Maxwell® RSC DNA FFPE Kit and the Maxwell® RSC Instrument (Promega, Madison, WI, USA) and quantified with the Qubit Fluorometer employing the Qubit dsDNA HS Assay Kit (Thermo Fisher Scientific, Waltham, MA, USA), according to the manufacturer´s protocol. Quality control polymerase chain reaction (PCR) was performed to determine the amplifiable DNA length [34]. Only cases with at least 100 base pairs (bp) amplifiable DNA were included for NGS analysis. Core biopsies were used instead, in cases where resection specimens had poor DNA quality (< 100 bp).

### Targeted NGS analysis

Targeted sequencing was performed using the Ion GeneStudio™S5 system (Thermo Fisher Scientific, Waltham, MA, USA). NGS analysis was performed using two panels − the Ion AmpliSeq *TP53* Community Panel and an Ion AmpliSeq™ custom *PIK3CA* panel from Thermo Fisher Scientific covering the entire coding regions of *TP53* (NM_000546.6) and *PIK3CA* (NM_006218.4), respectively (summarized in the Supplemental Table 1 and 2).

Amplicon library preparation and semiconductor sequencing were performed according to the manufacturer’s manuals using the Ion AmpliSeq Library Kit version 2.0, the Ion Library TaqMan Quantitation Kit on the LightCycler 480 (Roche, Basel, Switzerland), the Ion 540 Kit–Chef on the Ion Chef and the Ion 540 Chip Kit (Thermo Fisher Scientific). Output files were generated by Torrent Suite (version 5.16.1).

Variant calling was performed using the Ion Reporter Software (version 5.20.2.0; Thermo Fisher Scientific). Variants were visualized using the Integrative Genomics Viewer (IGV, version 2.16.2; Broad Institute, Cambridge, MA) to exclude panel-specific artifacts. For variant calling, standard settings were used (no allelic frequency detection limit threshold). Variants were considered at a variant allele frequency (VAF) of > 10% and a coverage of at least 91%. The National Center for Biotechnology Information single-nucleotide polymorphism database (dbSNP; including GnomAD, ExAC and TOPMED) was used to exclude SNPs.

### Statistical analysis

Statistical analysis was performed using JMP SAS 15.1.0 (SAS, Cary, NC, USA) and R v. 4.0.5 (RStudio Team (2022); RStudio: Integrated Development Environment for R.RStudio, PBC, Boston, MA URLhttp://www.rstudio.com/). Categorical variables were described using frequencies and percentages. Numerical variables were expressed as either mean and standard deviation (± SD) or median and interquartile range (IQR), according to the distribution of the data. Normality of distribution was assessed by testing kurtosis and skewness, as well as by QQ plots. Chi-square test was used to assess categorical variables, and kappa tests were performed to measure agreement. For survival analysis, ten cases were excluded due to a previous diagnosis of cancer. Disease-free survival (DFS) was defined as the time from diagnosis to the date of any disease recurrence (local, regional or distant), excluding death. Overall survival (OS) was defined as the time from diagnosis to the date of death from any cause or to the date of censoring at the last time the subject was known to be alive. DFS and OS curves were illustrated by Kaplan–Meier regression. A log-rank test was used for time-to-event outcomes.

Multivariate Cox regression analysis was performed to assess the clinical value of the *TP53* status on DFS and OS; hazard ratio (HR) and their 95% confidence intervals (95% CI) were calculated. Multivariable survival analyses were conducted using Cox proportional hazards regression modelling to assess the magnitude of impact while adjusting for well-known clinicopathological risk parameters (age, tumor stage, molecular subtypes). All *p*-values were two-sided, and *p* < 0.05 was considered statistically significant.

## Results

### Patient characteristics

Cases were categorized as follows: HR + HER2 − (85 cases; G1, 21; G2, 42; G3, 22), HER2 + (21 cases) and TN (25 cases). Five TNBC cases exhibited the morphology of carcinoma with apocrine differentiation. Based on their morphological features, the remaining 20 TNBC cases were classified in accordance with Weisman et al. as TNBC with prominent tumor infiltrating lymphocytes (TNBC TIL, 9 cases), TNBC with large central acellular zone (TNBC LCAZ, 6 cases) and TNBC not otherwise specified (TNBC NOS, 5 cases) [35]. The median age of the patients was 59 years (range, 26–89 years). The tumor median size was 18 mm (range, 3–103 mm). The T stage ranged from pT1-3. The numbers of different grading and nuclear grade per subgroup are shown in Table 1.

**Table 1:** Clinical and histopathological characteristics

	*n* (%)		
Total number	131 (100%)		
Median age (year; range)	59 (26–89)		
Site of involvement			
Right	75 (57.3%)		
Left	56 (42.7%)		
Median size of tumor (mm; range)	18 mm (3–103)		
Tumor stage			
1a	2 (1.5%)		
1b	15 (11.5%)		
1c	69 (52.7%)		
2	40 (30.5%)		
3	5 (3.8%)		
Therapy relevant groups			
HR + HER2 − G1	21 (16.0%)		
HR + HER2 − G2	42 (32.1%)		
HR + HER2 − G3	22 (16.8%)		
HER2 +	21 (16.0%)		
TN	25 (19.1%)		
Grading	**G1**	**G2**	**G3**
HR + HER2 −	21 (24.7%)	42 (49.4%)	22 (25.9%)
HER2 +	0 (0%)	9 (42.9%)	12 (57.1%)
TN	0 (0%)	4 (16.0%)	21 (84.0%)
Nuclear grade	**1**	**2**	**3**
HR + HER2 −	4 (4.7%)	44 (51.8%)	37 (43.5%)
HER2 +	0 (0%)	2 (9.5%)	19 (90.5%)
TN	0 (0%)	4 (16.0%)	21 (84.0%)

*HR* + hormone receptor positive, *HER2*** − **human epidermal growth factor receptor 2 negative, *TN* triple negative, *G1* histologic grade 1, *G2* histologic grade 2, *G3* histologic grade 3

### p53 immunohistochemistry

**Fig. 1:**
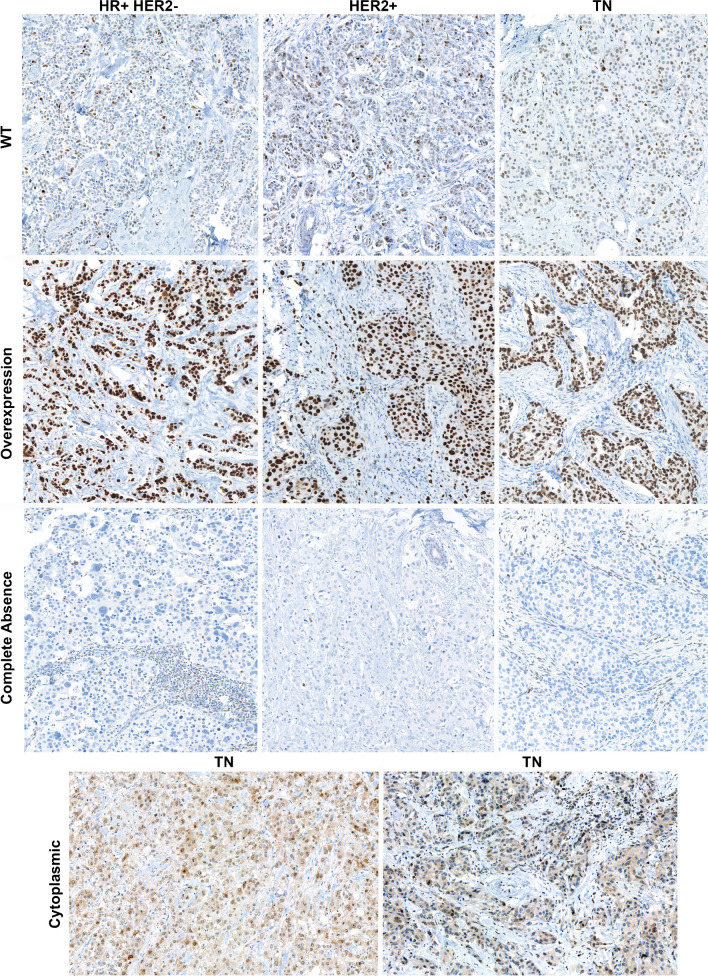
Examples of the different p53 staining patterns within the different subgroups: hormone receptor-positive and HER2-negative (HR + HER2 −), HER2-positive (HER2 +) and triple negative (TN). One example of wild-type (WT), overexpression (OE) and complete absence (CA) is shown per subgroup. The only two incidents of cytoplasmic (CY) staining were found in TN and are presented in the lower section

**Fig. 2:**
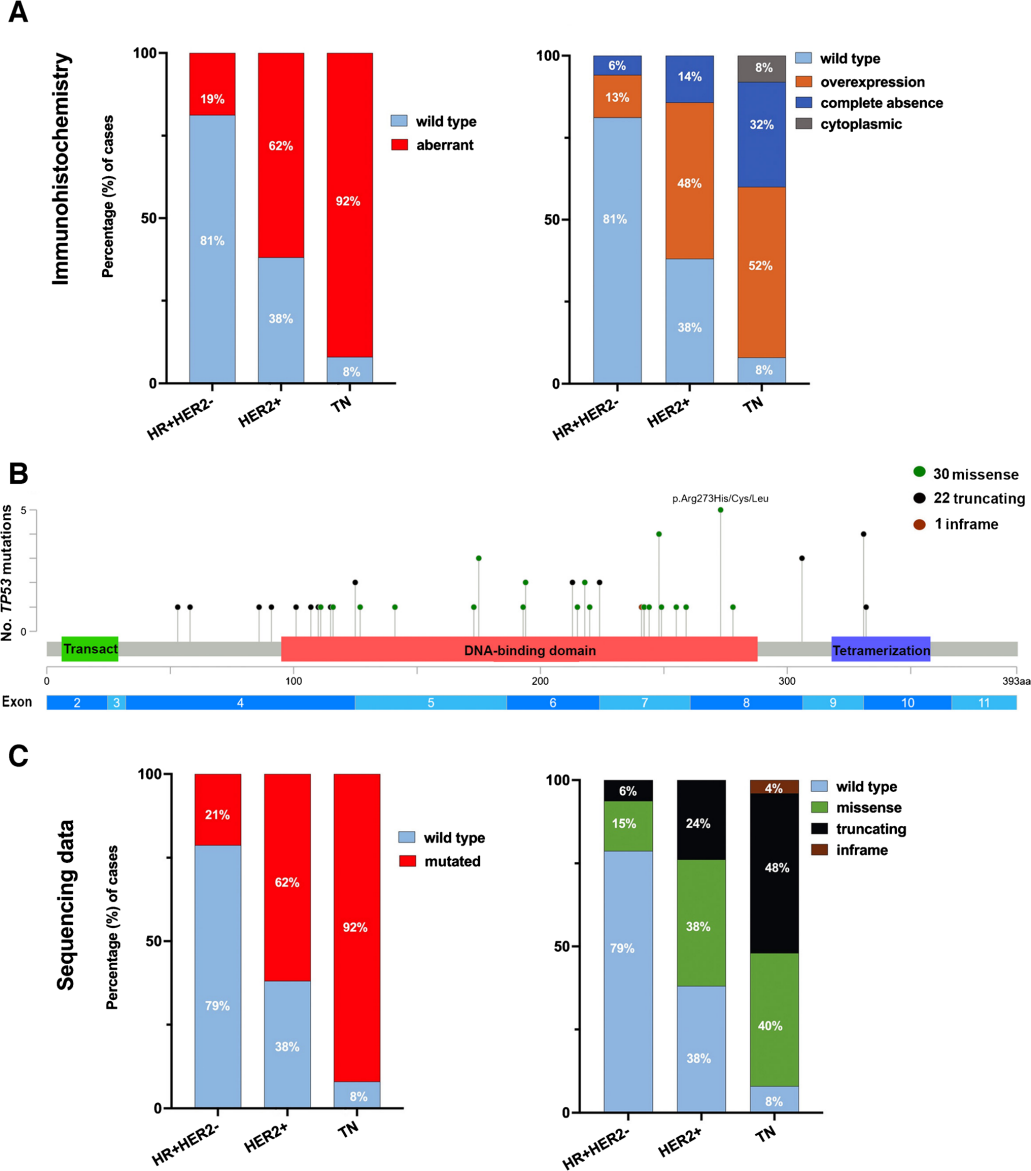
Evaluation of p53 by immunohistochemistry and targeted sequencing in different subtypes of breast cancer: HR + HER2 − , HER2 + and TN. **A** Graph plots depicting the percentages of cases with wild-type and aberrant staining of p53 by IHC (left side) and the different staining patterns per subgroup (right side). **B** Lollipop plot illustrating the localization and variant effect of mutations found in *TP53* representing the transactivation, tetramerization and DNA-binding domains. The number of mutations detected is represented on the ordinate and the variant effects are coded by the colours as shown in the legend. Transact: Transactivation domain. Created with cBioPortal (Cerami et al., Cancer Discov. 2012, and Gao et al., Sci. Signal. 2013). **C** Graph plots showing the percentages of cases with *TP53* mutations (left side) and their different variant effects per subgroup (right side)

The majority of cases exhibited a wild-type staining pattern (79/131, 60.3%) with variable numbers of positive tumor cells with heterogenous nuclear staining intensity. Aberrant staining patterns were detected in 52/131 cases (39.7%), interpreted as indicating the potential presence of a *TP53* mutation. The aberrant staining patterns were classified as OE, CA and CY (Fig. 1). OE refers to strong, homogenous and band-like staining of all well-fixed tumor cell nuclei, whereas CA is depicted as a total loss of staining in tumor cells, with preserved variable staining in non-tumor cell nuclei as an internal control, and CY describes a granular cytoplasmic staining with variable or missing nuclear staining. The highest prevalence of aberrant p53 staining was observed in TNBC (23/25, 92%, Fig. 2A left panel), while only 18.8% (16/85) were detected in HR + HER2 − (*p* < 0.001, Table 2). Strikingly, no aberrant staining was observed in the HR + HER2 − G1 subset. The most common aberrant staining pattern observed was OE (34/52, 65.4%), followed by CA (16/52, 30.7%), and CY (2/52, 3.8%). OE was the most common aberrant staining pattern in all subgroups, and only two TN cases showed CY staining (Fig. 2A right panel). The staining patterns between matched resection specimens and core biopsies showed complete agreement (124/124, 100%). In three cases, either the core biopsy or the resection specimen could not be evaluated due to an insufficient quantity of invasive tumor cells on the slide. In another four cases, no corresponding core biopsy was available. Therefore, in a total of seven cases, the staining pattern was analyzed in a single sample, either the core biopsy or the resection specimen.

**Table 2:** Comparison of p53 status evaluated by IHC and NGS with the different therapy-relevant groups

Therapy relevant groups	HR + HER2 −	HER2 +	TN	*p* value Xi^2^
p53 IHC aberrant	16/85 (18.8%)	13/21 (61.9%)	23/25 (92.0%)	0.001
p53 IHC staining pattern				
Overexpression	11/16 (68.8%)	10/13 (76.9%)	13/23 (56.5%)	0.001
Complete absence	5/16 (31.3%)	3/13 (23.1%)	8/23 (34.8%)	
Cytoplasmic	0/16 (0.0%)	0/13 (0.0%)	2/23 (8.7%)	
Presence of *TP53* mutation	17/80 (21.3%)	13/21 (61.9%)	23/25 (92.0%)	0.001
*TP53* variant effect				
Missense	12/17 (70.6%)	8/13 (61.5%)	10/23 (43.5%)	0.419
Truncating	5/17 (29.4%)	5/13 (38.5%)	12/23 (52.2%)	
Inframe	0/17 (0.0%)	0/13 (0.0%)	1/23 (4.3%)	

*HR* + hormone receptor positive, *HER2 − *human epidermal growth factor receptor 2 negative, *TN* triple negative, *G1* histologic grade 1, *G2* histologic grade 2, *G3* histologic grade 3, *IHC* immunohistochemistry

### Next-generation sequencing of *TP53* and *PIK3CA*

In total, *TP53* mutations were found in 53/126 amplifiable cases (42.1%). DNA extracted from the resection specimens was primarily used for NGS. If the DNA quality was insufficient, the corresponding core biopsy was used instead. In five samples, mutation status could not be determined due to poor DNA quality, both in resection specimens and in core biopsies. The *TP53* mutation rates observed in the different subtypes, namely HR + HER2 − , HER2 + and TNBC, were 21.3%, 61.9% and 92.0%, respectively. The two TNBC cases lacking a *TP53* mutation were observed to exhibit the morphological characteristics of carcinoma with apocrine differentiation. Consequently, the five TNBC cases with apocrine differentiation demonstrated a lower mutation rate (3/5, 60%) than the remaining 20 TNBC cases, which were subclassified as TNBC TIL, TNBC LCAZ and TNBC NOS with a 100% mutation rate, respectively.

Most of the *TP53* mutations (41/53, 77.4%) were found in the DNA-binding domain (Fig. 2B). The majority of the *TP53* mutations were classified as missense mutations (30/53, 56.6%), followed by truncating mutations (22/53, 41.5%) and one inframe mutation (1/53, 1.9%, Supplemental Table 3). Truncating mutations included nine splice site mutations, eight nonsense mutations, three frameshift deletions and two frameshift insertions. The TN subtype had the highest proportion of truncating mutations (Fig. 2C). Interestingly, missense mutations were more common in the HR + HER2 − and the HER2 + subgroups (Table 2).

In addition to the NGS analysis of *TP53*, we also sequenced *PIK3CA*. In total, *PIK3CA* mutations were found in 54/126 amplifiable cases (42.9%). Almost half of these mutations were found in hotspot p.His1047Arg/Leu (26/54, Supplemental Fig. 1). The majority of mutations identified, 50/54 (92.6%), were classified as missense mutations and only four were classified as inframe mutations (7.4%).

### Concordance between p53 immunostaining and *TP53* mutational status

**Fig. 3:**
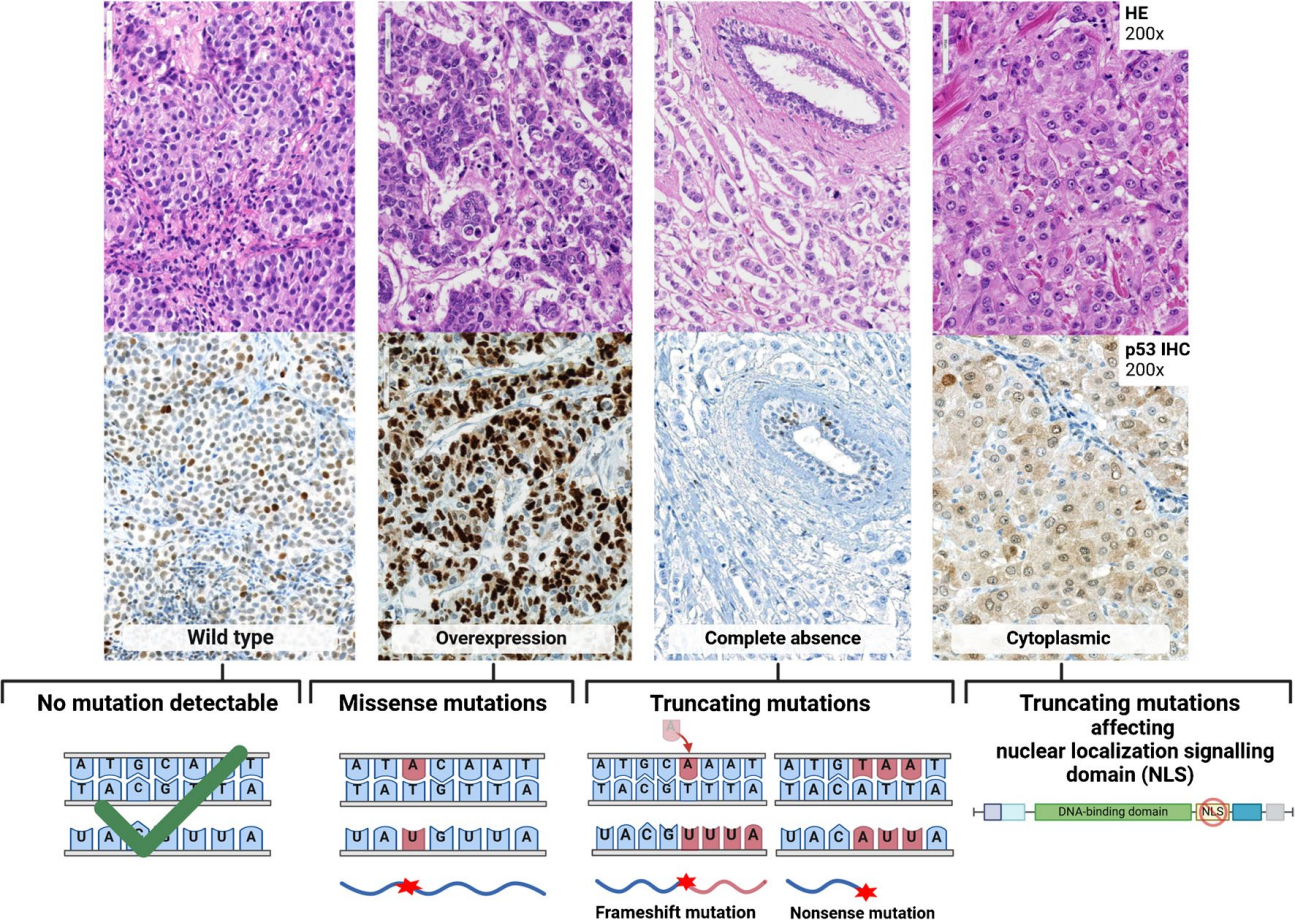
Overview of different p53 staining patterns with corresponding H&E staining, indicating the type of associated *TP53* mutation. The wild-type pattern was associated with no detectable mutations, whereas the aberrant patterns of overexpression, complete absence and cytoplasmic staining were associated with missense mutations, truncating mutations and truncating mutations affecting the nuclear localization domain, respectively. Created with BioRender.com

Overall, the comparison of the protein and genotype level showed a sensitivity of 96.2% and a specificity of 100% for the immunohistochemical detection of the *TP53* mutation. Furthermore, there was a significant level of agreement between missense mutations and OE, as well as between truncating mutations and CA (Cohen’s κ 73% and 76%, respectively). NGS analysis revealed that the two cases exhibiting CY staining in IHC had truncating mutations both within the nuclear localization signalling domain of p53 (p.Arg306Ter and p.X306_splice), likely resulting in mislocalization of the protein. Only two HR + HER2 − cases of grade 2 were false negatives by IHC, both carrying missense mutations (p.Ser116Cys and p.Arg175His, Fig. 3).

### Histopathological features of *TP53*-mutated cases and association with *PIK3CA* mutational status

**Fig. 4:**
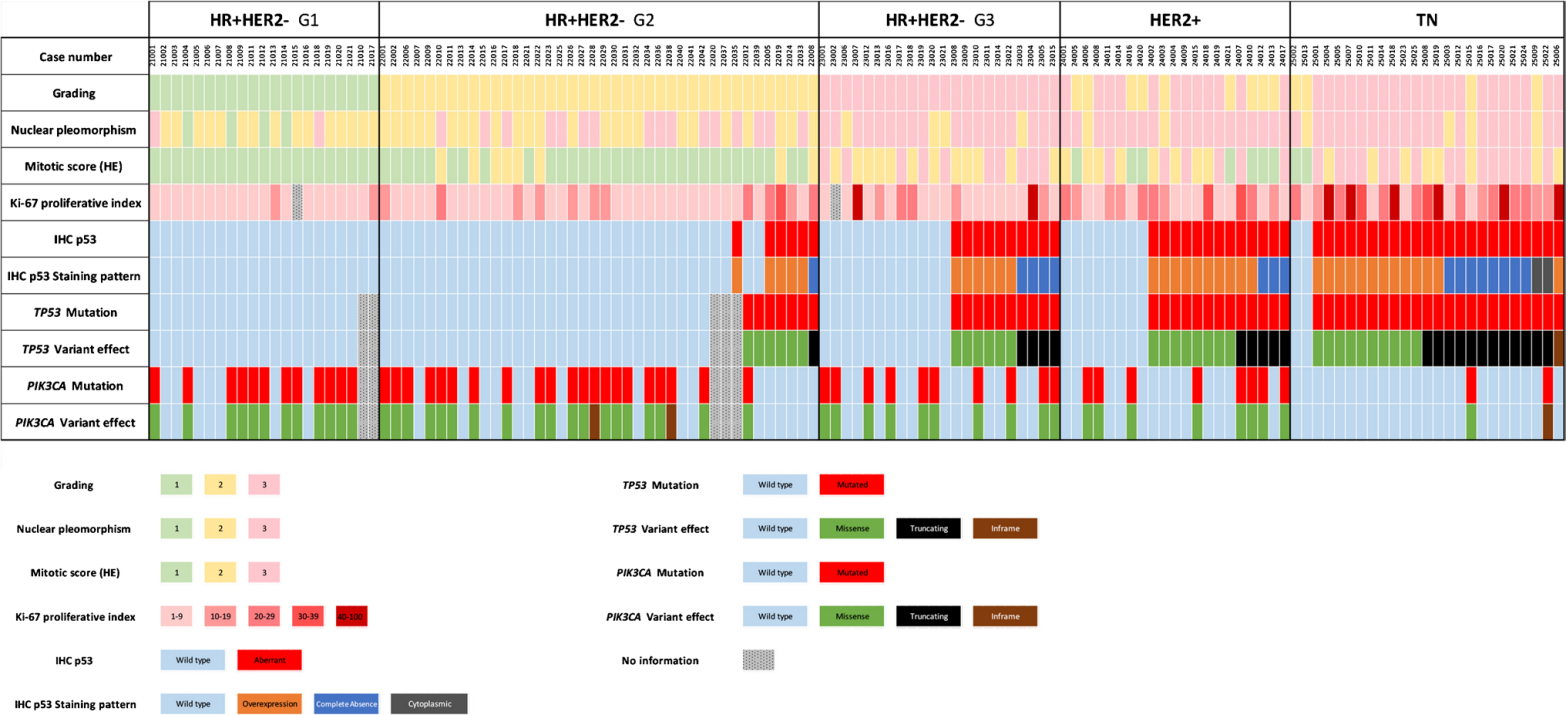
Heatmap showing mutational status regarding *TP53* and *PIK3CA* and the histopathological characteristics. Cases were consecutively collected within these five subgroups (HR + HER2 − G1, HR + HER2 − G2, HR + HER2 − G3, HER2 + , TN). The cases were grouped according to the following criteria, with decreasing priority: *TP53* mutation versus wild-type (NGS), *TP53* variant effect (NGS), p53 aberrant versus wild-type (IHC) and p53 staining patterns (IHC). The legend provides the colour coding for the various functions

*TP53*-mutated cases exhibited higher nuclear pleomorphism (*p* < 0.001) and grade (G3, 73.6%; G2, 26.4%; G1, 0%) compared to wild-type cases (G3, 21.9%; G2, 52.1%; G1, 26.0%) (*p* < 0.001, Fig. 4). Similar trends were evident in HR + HER2 − (*p* < 0.001 and *p* < 0.04). *TP53* mutations were also linked to a high Ki-67 proliferation index (*p* < 0.001). *TP53* and *PIK3CA* mutations showed an inverse correlation. In contrast, the subgroups of HR + HER2 − , HER2 + and TNBC displayed *PIK3CA* mutations in 55.0%, 38.1% and 8.0% of cases, respectively. Notably, there was a strong association between mutant *TP53* and wild-type *PIK3CA* (*p* < 0.001).

### Prognostic impact of *TP53* mutational status and aberrant immunostaining

The statistical analysis of DFS and mutation status is summarized in Supplemental Fig. 2. By Kaplan–Meier analysis of all patients, p53 mutation/expression status was not statistically associated with DFS, showing comparable DFS between *TP53*-mutated and wild-type cases, as well as between p53 aberrant and wild-type staining (respectively, Supplemental Fig. 2A). By univariate analysis, p53 mutation and aberrant staining were correlated with HR values of 2.6 and 2.9, respectively, but this was not statistically significant (Supplemental Fig. 2 B). A slight disparity in DFS was observed when comparing only the p53 mutation/expression status of cases classified as HR + HER2 − . However, this difference was not statistically significant (Supplemental Fig. 2C). There was no significant difference in OS between *TP53*-mutated and wild-type cases (Supplemental Fig. 3).

## Discussion

Our study demonstrates that a comprehensive evaluation of p53 staining patterns, rather than an arbitrary cutoff, shows a high sensitivity (96%) and specificity (100%) in predicting the presence and type of *TP53* mutations in BC of NST. These encouraging results indicate that p53 IHC could serve as a reliable surrogate marker to identify patients at higher risk for resistance to endocrine therapy and may be used as a cost-effective screening tool. Furthermore, we observed a significant association of *TP53* mutations with high tumor grade, high nuclear grade, proliferative activity and TN status. Within the HR + HER2 − group, we observed 21% cases with mutant *TP53*, with a clear preference for high-grade carcinomas. Moreover, we identified a negative association with *PIK3CA* mutations, suggesting distinct tumor clusters with activation of alternative pathways.

Previous clinical studies attempting to define p53 staining as a surrogate to predict mutations in BC have relied on the OE and/or CA staining pattern, which have yielded different cut-off values, ranging from 10 to 50% positive cells, or CA only [4, 6, 16, 18, 22]. In a previous study, a cutoff of 35% was described when assessing the OE pattern, resulting in a sensitivity of 65% and a specificity of 95%. However, cases with a CA pattern were not included, as this pattern was considered insignificant in cases of HR + HER2 − [22]. Nevertheless, in our study, almost one-third of HR + HER2 − cases exhibited CA staining, thereby emphasizing the significance of employing a combination of staining patterns during the IHC assessment. In the present study, we propose the implementation of an IHC algorithm analogous to that previously described for ovarian and endometrial cancer [17, 28, 32]. We validated aberrant pattern interpretation of p53 IHC by targeted sequencing of the entire *TP53* gene in a well-defined series of BC of NST representing different therapy-relevant subtypes. This resulted in a strong agreement between the staining pattern and the type of mutation.

Most cases carrying a *TP53* mutation showed OE staining, predominantly associated with missense mutations. CA was observed in cases with truncating mutations, while CY staining was attributed to alterations in the nuclear localization signal. Specifically, the both truncating mutations p.Arg306Ter and p.X306_splice corresponding to the CY staining and observed in TN were found within the nuclear localization signalling domain (305–322 aa) of p53 which might lead to a cytoplasmic accumulation of the protein, as previously described by Köbel et al. [17]. To the best of our knowledge, this is the first study to describe the CY pattern of p53 IHC in BC and to match this pattern with the underlying genetic alterations in the nuclear localization signalling domain of the *TP53* gene.

In 2/53 cases with *TP53* mutations, an aberrant IHC staining pattern could not be identified. Among these two false-negatives cases, one case contained the missense mutation p.Ser116Cys, which is considered a variant of unknown significance (VUS) according to the most recent updates in the respective databases. Supposedly, the mutation still maintains a partial wild-type function, which may lead to a wild-type-like staining pattern and might not have an impact on the cellular biology of the tumor cells. The second case showed the missense mutation p.Arg175His, which is a well-documented hotspot mutation, which was detected by aberrant OE staining in another case within our cohort. The IHC staining was re-evaluated using both specimens, the core biopsy and the resection specimen. In this case, the discrepant result could not be attributed to fixation artefacts.

Given that the cases in this cohort did not undergo neoadjuvant therapy, the interval between the removal of the core biopsy and the resection specimens was relatively brief. Consequently, the tissue of both samples is considered to exhibit a high degree of biological similarity, which allows for the comparison of the samples and provides a satisfactory explanation for the 100% agreement of the staining patterns between the core biopsies and the resection specimens. This approach allowed us to demonstrate that both core biopsies and resection specimens can be employed for the evaluation of p53 status.

The frequencies of aberrant p53 expression in our study, affecting 19% of HR + HER2 − , 62% of HER2 + cases and 92% of TNBC were consistent with the documented mutation rates for the different subtypes of BC [27]. Additionally, in line with recent findings, TNBC cases with apocrine differentiation demonstrated a lower *TP53* mutation rate than the overall TNBC cohort [35]. Evaluation of *TP53* status in HR + HER2 − cases may be of particular interest for future treatment decisions, especially in cases with favourable or intermediate pathological features. In recent studies, in addition to tumor stage, grading and hormone-receptor status, a variety of additional markers have been evaluated, including the 21-gene recurrence score, the PAM50 risk of recurrence score and changes in Ki-67 after short-term endocrine therapy [10, 24, 25, 29, 30]. In this context, a rapid evaluation of *TP53* status may assist in the establishment of an additional risk category in HR + HER2 − .The reliable and widely available strategy with pattern analysis in IHC of this study might help to evaluate larger clinical studies. This may be able to provide the necessary clinical confirmation from larger retrospective analysis or to plan future prospective trials, which include the affordable p53 protein status as a secondary parameter in addition to gene expression assays. Moreover, approaches targeting *TP53* mutations that were formerly deemed undruggable are now being subjected to rigorous investigation, with some already undergoing clinical trials [14, 20, 23]. Consequently, *TP53* mutation status may emerge as a pivotal predictive marker for therapies tailored to *TP53*-mutated BC.

The prognostic impact of aberrant p53 IHC with a bimodal pattern, similar to our strategy but lacking the CY pattern, has been previously analyzed [3]. Boyle et al. demonstrated that p53 aberrant staining is significantly associated with shorter OS and DFS. Interestingly, the strongest effect on survival was observed within the group of HR + HER2 − , independently of the results in the TNBC cases. In our study, no differences in DFS and OS were identified in cases of aberrant p53 status in the complete cohort and HR + HER2 − cases. This may be due to the limited number of cases in the survival analysis and the exclusion of patients with neoadjuvant treatment, a relevant segment of high-risk HR + HER2 − cases, which were not part of the study.

In addition to the relatively small sample size of our cohort and the focus on BC of NST, some limitations may be acknowledged in our study including the potential for tissue degradation due to the age of our samples, which exceeded 10 years. To ensure the integrity of the DNA during sequencing analysis, 5/131 samples with less than 100 bp in the quality control PCR were excluded. Additionally, IHC evaluation was limited to areas on the slides exhibiting adequately fixed tissue.

In previous studies, *TP53* mutations were associated with poor prognosis of primary BC and predicted potential endocrine resistance of the HR + HER2 − subtype, making a suitable screening tool desirable. In our study, IHC for p53 with interpretation of specific aberrant staining patterns could reliably identify patients with mutant *TP53* in a simple and affordable manner. The staining patterns matched the expected types of mutations in NGS, confirming the validity of this approach. Therefore, this strategy might facilitate future studies to evaluate the impact of *TP53* mutations on the benefit of specific therapeutic strategies. Eventually, like in endometrial carcinoma, p53 IHC might become part of the routine diagnostic panel in BC.

## Supplementary Information

Below is the link to the electronic supplementary material.

Supplementary file1 (DOCX 1679 KB)

## Data Availability

The data sets used and analyzed during this study are available from the corresponding author on reasonable request.

## Declarations

### Statement

During the preparation of this work the authors used *DeepL Write* in order to check grammar and in part to improve readability. After using this tool, the authors reviewed and edited the content as needed and take full responsibility for the content of the publication.
