# Supplementary material for: Aberrant p53 immunostaining patterns in breast carcinoma of no special type strongly correlate with presence and type of TP53 mutations
Source: Virchows Arch. 2024 Aug 27;485(4):631–42. doi: 10.1007/s00428-024-03897-3 (PMC11522169; doi:10.1007/s00428-024-03897-3)
Supplement: Supplementary file 1 — Supplementary file1 (DOCX 1679 KB) [file 428_2024_3897_MOESM1_ESM.docx]

**Supplemental Table 1. Overview of the Ion Research panel *TP53* (1.28 kb)**

| **Target ID** | **Position in Chr17** | **No. of**  **Amplicons** | **Target**  **Bases** | **Covered**  **Bases** | **Missed**  **Bases** | **Coverage [%]** |
| --- | --- | --- | --- | --- | --- | --- |
| 1 | 7572927-7573008 | 2 | 82 | 82 | 0 | 100 |
| 2 | 7573927-7574033 | 2 | 107 | 107 | 0 | 100 |
| 3 | 7576537-7576584 | 1 | 48 | 48 | 0 | 100 |
| 4 | 7576625-7576657 | 1 | 33 | 33 | 0 | 100 |
| 5 | 7576853-7576926 | 2 | 74 | 74 | 0 | 100 |
| 6 | 7577019-7577155 | 2 | 137 | 137 | 0 | 100 |
| 7 | 7577499-7577608 | 2 | 110 | 110 | 0 | 100 |
| 8 | 7578177-7578289 | 2 | 113 | 113 | 0 | 100 |
| 9 | 7578371-7578554 | 3 | 184 | 184 | 0 | 100 |
| 10 | 7579302-7579590 | 4 | 289 | 289 | 0 | 100 |
| 11 | 7579700-7579721 | 1 | 22 | 22 | 0 | 100 |
| 12 | 7579839-7579912 | 1 | 74 | 74 | 0 | 100 |
| 13 | 7578112-7578118 | 1 | 7 | 7 | 0 | 100 |
| Target ID: refers to specific region in gene e.g. exons, Position in Chr17: Start and end of target region in chromosome 17, No. of Amplicons: number of amplicons used to cover target exon, Target Bases: total number of bases targeted within target region, Covered Bases: number of bases covered by amplicon reads, Missed Bases: number of bases not covered by amplicon reads, Coverage: ratio of number of covered bases and target bases in percentage (%). | | | | | | |

**Supplemental Table 2.** Overview of the designed Ion AmpliSeq Custom Panel *PIK3CA* (4.75 kb).

| **Exon no.** | **Position in Chr3** | **No. of**  **Amplicons** | **Total**  **Bases** | **Covered**  **Bases** | **Missed**  **Bases** | **Coverage [%]** |
| --- | --- | --- | --- | --- | --- | --- |
| 433394 | 178916608-178916970 | 5 | 362 | 362 | 0 | 100 |
| 433399 | 178917472-178917692 | 3 | 220 | 220 | 0 | 100 |
| 433390 | 178919072-178919333 | 3 | 261 | 261 | 0 | 100 |
| 433398 | 178921326-178921582 | 4 | 256 | 256 | 0 | 100 |
| 433403 | 178922285-178922381 | 2 | 96 | 96 | 0 | 100 |
| 433401 | 178927377-178927493 | 2 | 116 | 116 | 0 | 100 |
| 433400 | 178927968-178928131 | 2 | 163 | 163 | 0 | 100 |
| 433395 | 178928213-178928358 | 2 | 145 | 145 | 0 | 100 |
| 433396 | 178935992-178936127 | 1 | 135 | 111 | 24 | 82.2 |
| 433389 | 178936978-178937070 | 1 | 92 | 92 | 0 | 100 |
| 433388 | 178937353-178937528 | 2 | 175 | 156 | 19 | 89.1 |
| 433387 | 178937731-178937845 | 1 | 114 | 87 | 27 | 76.3 |
| 433402 | 178938768-178938950 | 2 | 182 | 182 | 0 | 100 |
| 433393 | 178941863-178941980 | 3 | 117 | 117 | 0 | 100 |
| 433392 | 178942482-178942614 | 2 | 132 | 132 | 0 | 100 |
| 433391 | 178943744-178943833 | 1 | 89 | 89 | 0 | 100 |
| 433385 | 178947054-178947235 | 3 | 181 | 181 | 0 | 100 |
| 433384 | 178947786-178947914 | 2 | 128 | 128 | 0 | 100 |
| 433397 | 178948007-178948169 | 2 | 162 | 162 | 0 | 100 |
| 433386 | 178951876-178952157 | 3 | 281 | 281 | 0 | 100 |
| Target ID: refers to specific region in gene e.g. exons, Position in Chr17: Start and end of target region in chromosome 17, No. of Amplicons: number of amplicons used to cover target exon, Target Bases: total number of bases targeted within target region, Covered Bases: number of bases covered by amplicon reads, Missed Bases: number of bases not covered by amplicon reads, Coverage: ratio of number of covered bases and target bases in % | | | | | | |

**Supplemental Table 3. All mutations detected in *TP53* and *PIK3CA* in this study.**

| **Sample ID** | **Gene** | **cDNA** | **Protein change** | **Mutation type** |
| --- | --- | --- | --- | --- |
| 22005 | *TP53* | c.764T>G | p.Ile255Ser | missense |
| 22008 | *TP53* | c.173delC | p.Pro58GlnfsTer65 | truncating |
| 22012 | *TP53* | c.524G>A | p.Arg175His | missense |
| 22019 | *TP53* | c.581T>G | p.Leu194Arg | missense |
| 22024 | *TP53* | c.776A>T | p.Asp259Val | missense |
| 22033 | *TP53* | c.743G>A | p.Arg248Gln | missense |
| 22039 | *TP53* | c.347C>G | p.Ser116Cys | missense |
| 23003 | *TP53* | c.375+5G>C | p.X125_splice | truncating |
| 23004 | *TP53* | c.257_279delCACCAGCCCCCTCCTGGCCCCTG | p.Ala86ValfsTer55 | truncating |
| 23005 | *TP53* | c.273G>A | p.Trp91Ter | truncating |
| 23008 | *TP53* | c.581T>G | p.Leu194Arg | missense |
| 23009 | *TP53* | c.817C>T | p.Arg273Cys | missense |
| 23010 | *TP53* | c.743G>A | p.Arg248Gln | missense |
| 23011 | *TP53* | c.818G>T | p.Arg273Leu | missense |
| 23014 | *TP53* | c.380C>T | p.Ser127Phe | missense |
| 23015 | *TP53* | c.637C>T | p.Arg213Ter | truncating |
| 23022 | *TP53* | c.659A>G | p.Tyr220Cys | missense |
| 24002 | *TP53* | c.747G>T | p.R249S | missense |
| 24003 | *TP53* | c.818G>A | p.Arg273His | missense |
| 24004 | *TP53* | c.818G>A | p.Arg273His | missense |
| 24007 | *TP53* | c.994-1G>T | p.X331_splice | truncating |
| 24009 | *TP53* | c.731G>T | p.Gly244Val | missense |
| 24010 | *TP53* | c.994_1012delATCCGTGGGCGTGAGCGCT | p.Ile332SerfsTer7 | truncating |
| 24012 | *TP53* | c.919+1G>T | p.X306_splice | truncating |
| 24013 | *TP53* | c.672+2T>G | p.X224_splice | truncating |
| 24015 | *TP53* | c.653T>G | p.Val218Gly | missense |
| 24017 | *TP53* | c.301A>T | p.Lys101Ter | truncating |
| 24018 | *TP53* | c.644G>T | p.Ser215Ile | missense |
| 24019 | *TP53* | c.743G>A | p.Arg248Gln | missense |
| 24021 | *TP53* | c.742C>T | p.Arg248Trp | missense |
| 25001 | *TP53* | c.651_653delinsCGG | p.Val218Gly | missense |
| 25003 | *TP53* | c.993+1G>C | p.X331_splice | truncating |
| 25004 | *TP53* | c.725G>T | p.Cys242Phe | missense |
| 25005 | *TP53* | c.578A>G | p.His193Arg | missense |
| 25006 | *TP53* | c.722_724del | p.Ser241del | Inframe |
| 25007 | *TP53* | c.332T>A | p.Leu111Gln | missense |
| 25008 | *TP53* | c.376-2A>T | p.X125_splice | truncating |
| 25009 | *TP53* | c.916C>T | p.Arg306Ter | truncating |
| 25010 | *TP53* | c.422G>A | p.Cys141Tyr | missense |
| 25011 | *TP53* | c.818G>A | p.Arg273His | missense |
| 25012 | *TP53* | c.322_341dup | p.His115ValfsTer15 | truncating |
| 25014 | *TP53* | c.524G>A | p.Arg175His | missense |
| 25015 | *TP53* | c.159G>A | p.Trp53Ter | truncating |
| 25016 | *TP53* | c.672+2T>C | p.X224_splice | truncating |
| 25017 | *TP53* | c.993+1G>T | p.X331_splice | truncating |
| 25018 | *TP53* | c.524G>A | p.Arg175His | missense |
| 25019 | *TP53* | c.993+2delT | p.X331_splice | truncating |
| 25020 | *TP53* | c.329dup | p.Arg110ProfsTer39 | truncating |
| 25021 | *TP53* | c.637C>T | p.Arg213Ter | truncating |
| 25022 | *TP53* | c.920-1G>T | p.X306_splice | truncating |
| 25023 | *TP53* | c.832C>T | p.Pro278Ser | missense |
| 25024 | *TP53* | c.319_320insAAAAAAAAGCCTACCAGGGCAGCT | p.Tyr107Ter | truncating |
| 25025 | *TP53* | c.517G>A | p.Val173Met | missense |
| 21001 | *PIK3CA* | c.3140A>G | p.His1047Arg | missense |
| 21004 | *PIK3CA* | c.3140A>T | p.His1047Leu | missense |
| 21008 | *PIK3CA* | c.3140A>G | p.His1047Arg | missense |
| 21009 | *PIK3CA* | c.1633G>A | p.Glu545Lys | missense |
| 21011 | *PIK3CA* | c.1035T>A | p.Asn345Lys | missense |
| 21012 | *PIK3CA* | c.1633G>A | p.Glu545Lys | missense |
| 21014 | *PIK3CA* | c.1625A>C | p.Glu542Ala | missense |
| 21015 | *PIK3CA* | c.3145G>C | p.Gly1049Arg | missense |
| 21018 | *PIK3CA* | c.1633G>A | p.Glu545Lys | missense |
| 21019 | *PIK3CA* | c.3140A>G | p.His1047Arg | missense |
| 21020 | *PIK3CA* | c.3140A>G | p.His1047Arg | missense |
| 21021 | *PIK3CA* | c.3140A>G | p.His1047Arg | missense |
| 22001 | *PIK3CA* | c.1624G>A | p.Glu542Lys | missense |
| 22002 | *PIK3CA* | c.1035T>A | p.Asn345Lys | missense |
| 22004 | *PIK3CA* | c.1259_1264delGTCCAT | p.Cys420_Pro421del | inframe |
| 22006 | *PIK3CA* | c.3140A>G | p.His1047Arg | missense |
| 22009 | *PIK3CA* | c.3140A>G | p.His1047Arg | missense |
| 22010 | *PIK3CA* | c.3140A>T | p.His1047Leu | missense |
| 22011 | *PIK3CA* | c.3140A>T | p.His1047Leu | missense |
| 22012 | *PIK3CA* | c.3127A>G | p.Met1043Val | missense |
| 22014 | *PIK3CA* | c.3140A>G | p.His1047Arg | missense |
| 22017 | *PIK3CA* | c.3140A>G | p.His1047Arg | missense |
| 22022 | *PIK3CA* | c.1633G>A | p.Glu545Lys | missense |
| 22023 | *PIK3CA* | c.3140A>G | p.His1047Arg | missense |
| 22026 | *PIK3CA* | c.3140A>G | p.His1047Arg | missense |
| 22027 | *PIK3CA* | c.3140A>G | p.His1047Arg | missense |
| 22028 | *PIK3CA* | c.1253_1264delAACACTGTCCAT | p.Glu418_Leu422delinsVal | inframe |
| 22029 | *PIK3CA* | c.1633G>A | p.Glu545Lys | missense |
| 22030 | *PIK3CA* | c.3145G>C | p.Gly1049Arg | missense |
| 22031 | *PIK3CA* | c.1633G>A | p.Glu545Lys | missense |
| 22034 | *PIK3CA* | c.1624G>A | p.Glu542Lys | missense |
| 22036 | *PIK3CA* | c.3140A>G | p.His1047Arg | missense |
| 22038 | *PIK3CA* | c.1340_1366del | p.Pro447_Leu455del | inframe |
| 22042 | *PIK3CA* | c.1633G>A | p.Glu545Lys | missense |
| 23001 | *PIK3CA* | c.1633G>A | p.Glu545Lys | missense |
| 23002 | *PIK3CA* | c.1035T>A | p.Asn345Lys | missense |
| 23005 | *PIK3CA* | c.1633G>A | p.Glu545Lys | missense |
| 23010 | *PIK3CA* | c.3062A>G | p.Tyr1021Cys | missense |
| 23012 | *PIK3CA* | c.3140A>G | p.His1047Arg | missense |
| 23015 | *PIK3CA* | c.3140A>G | p.His1047Arg | missense |
| 23016 | *PIK3CA* | c.3140A>G | p.His1047Arg | missense |
| 23019 | *PIK3CA* | c.3140A>G | p.His1047Arg | missense |
| 23020 | *PIK3CA* | c.1633G>A | p.Glu545Lys | missense |
| 23022 | *PIK3CA* | c.1633G>A | p.Glu545Lys | missense |
| 24006 | *PIK3CA* | c.1633G>A | p.Glu545Lys | missense |
| 24007 | *PIK3CA* | c.3140A>G | p.His1047Arg | missense |
| 24008 | *PIK3CA* | c.3140A>G | p.His1047Arg | missense |
| 24010 | *PIK3CA* | c.1637A>G | p.Gln546Arg | missense |
| 24012 | *PIK3CA* | c.3140A>T | p.His1047Leu | missense |
| 24015 | *PIK3CA* | c.3140A>G | p.His1047Arg | missense |
| 24016 | *PIK3CA* | c.1258T>C | p.Cys420Arg | missense |
| 24017 | *PIK3CA* | c.3140A>T | p.His1047Leu | missense |
| 25015 | *PIK3CA* | c.3140A>G | p.His1047Arg | missense |
| 25022 | *PIK3CA* | c.1358_1363delAAGATT | p.Glu453_Leu  455delinsVal | inframe |

**Supplemental Figure 1.**


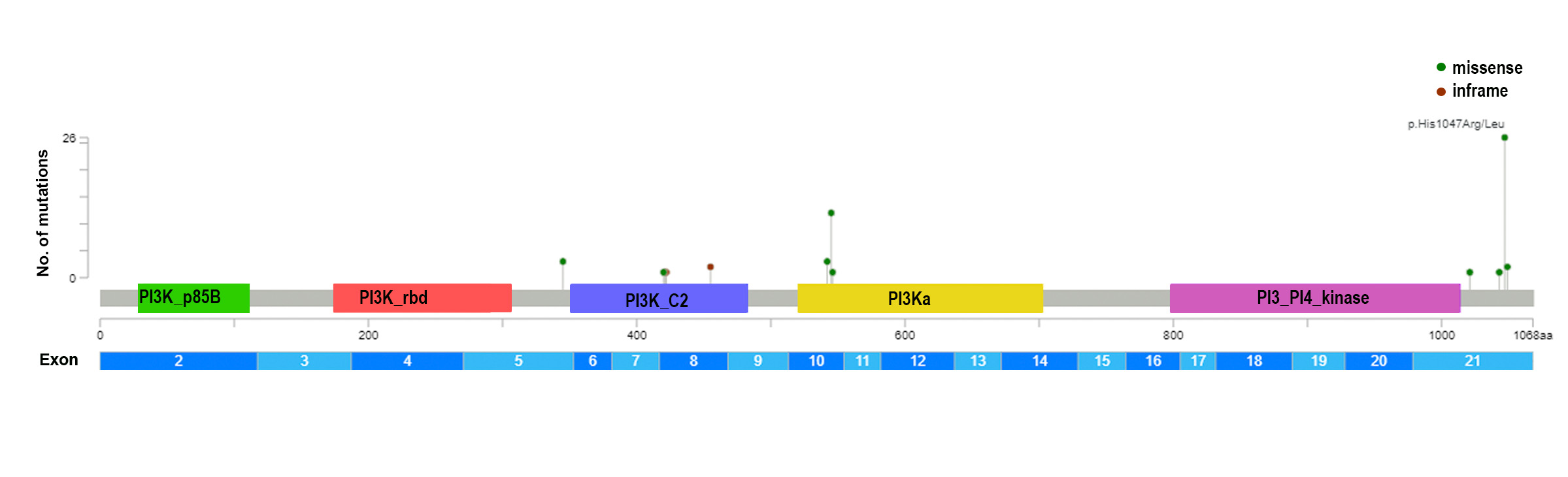


PI3K_p85B Phosphoinositide 3-kinase p85-binding domain, PI3K_rbd: Phosphoinositide 3-kinase ras-binding domain, PI3K_C2: Phosphoinositide 3-kinase C2, PI3Ka: Phosphoinositide 3-kinase accessory domain (PIK domain) and PI3_PI4_kinase: 4-kinase domain.

**
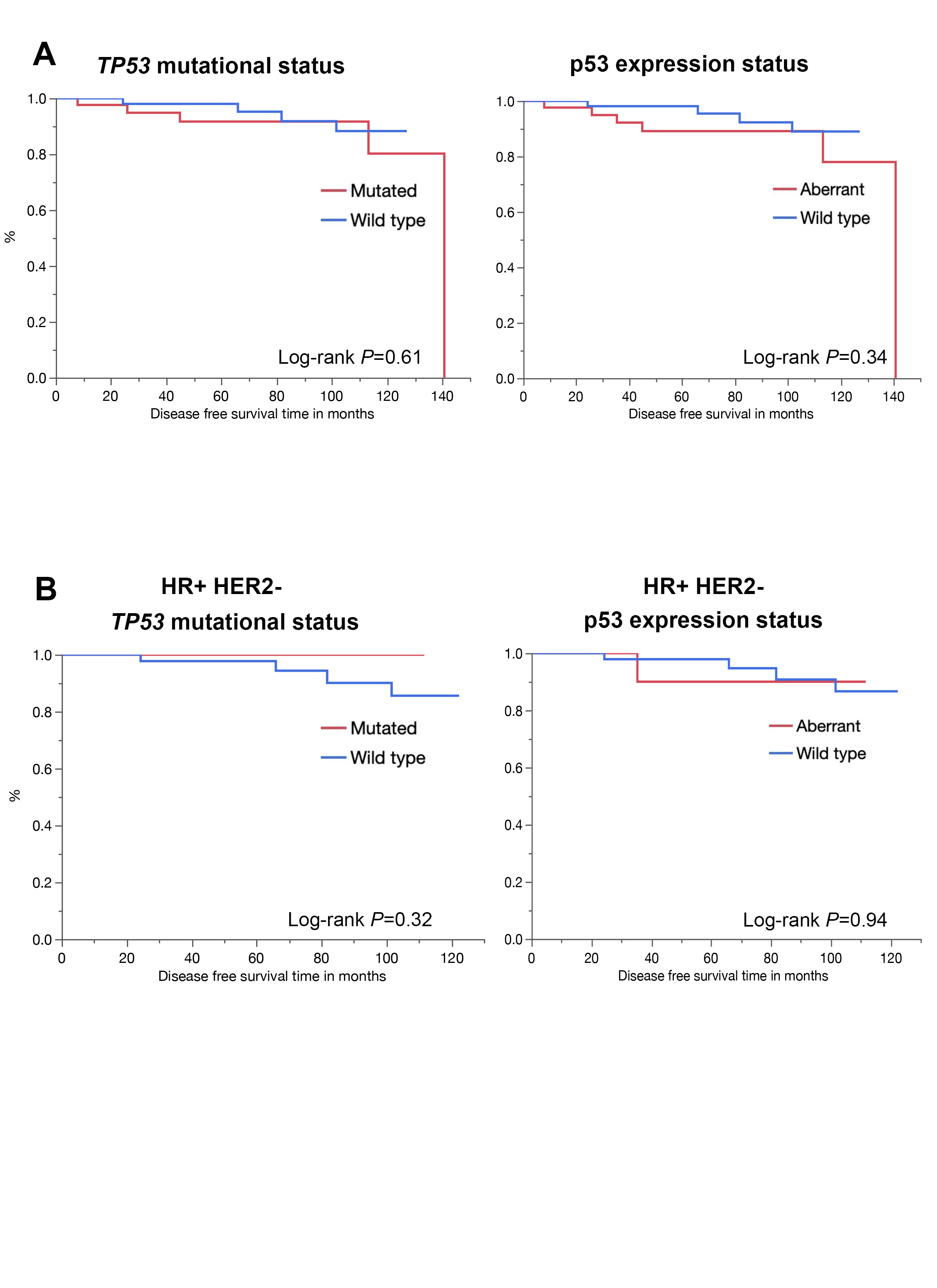
**

**Supplemental Figure 2. Kaplan Meyer analysis of p53 status and disease-free survival of cases with available follow up.** Kaplan-Meier curves illustrating the disease-free survival (DFS) in *TP53* mutated and wild type cases analyzed by targeted sequencing, as well as between p53 aberrant and wild type staining using IHC. The upper panel shows all the cases, and the lower panel refers to the cases within the HR+HER2- group.

**
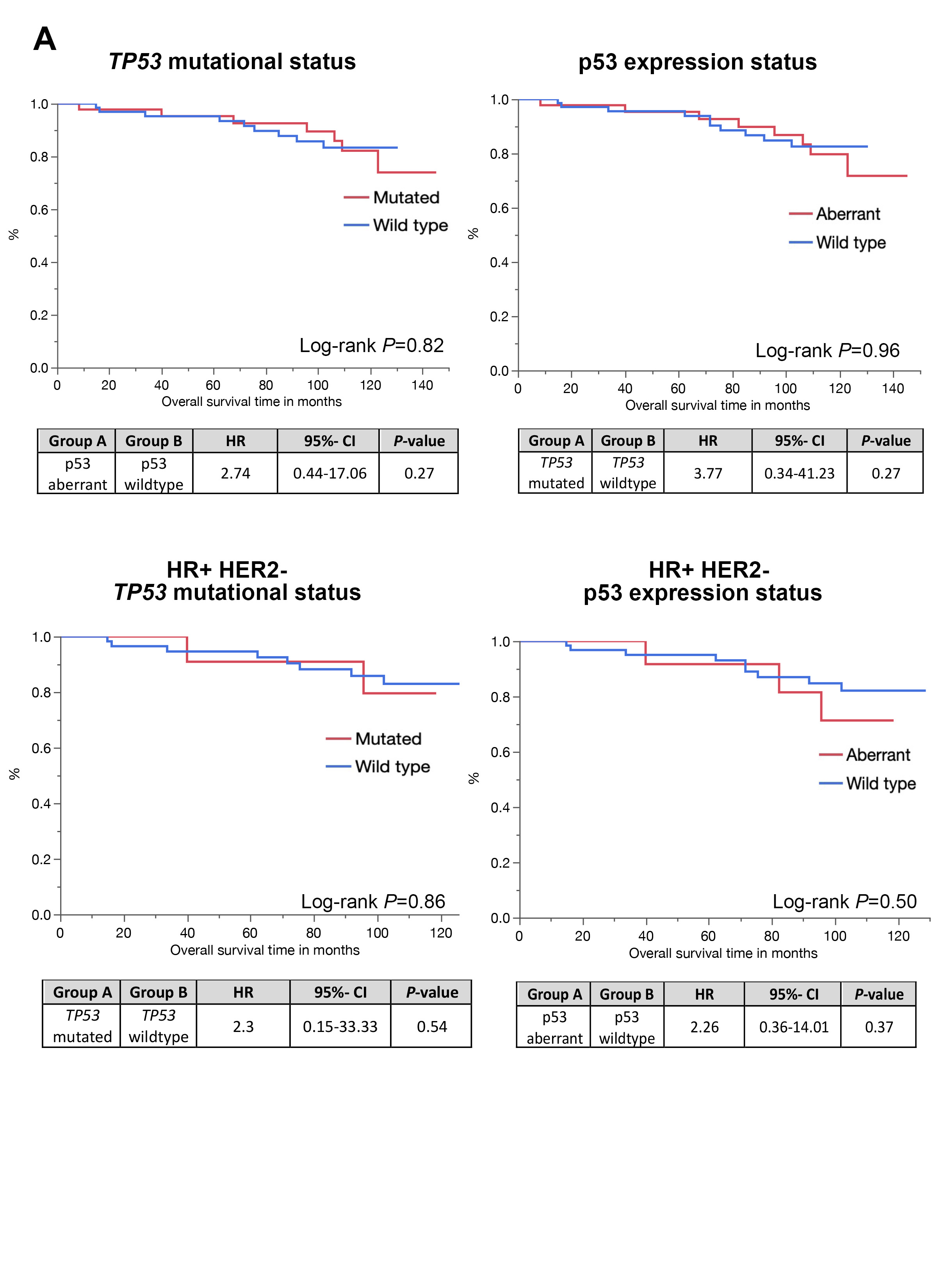
**

**Supplemental Figure 3. Kaplan Meyer and multivariate analysis of p53 status and overall survival of cases with available follow up.** Kaplan-Meier curves illustrating the overall survival (OS) in *TP53* mutated and wild type cases analyzed by targeted sequencing, as well as between p53 aberrant and wild type staining using IHC. The upper panel shows all the cases, and the lower panel refers to the cases within the HR+HER2- group.
